# A case of malignant solitary fibrous tumor of the prostatic urethra

**DOI:** 10.1259/bjrcr.20180034

**Published:** 2018-06-01

**Authors:** Yoshikazu Tanaka, Atsushi Nakamoto, Yuki Inada, Yoshifumi Narumi, Yoshinobu Hirose, Haruhito Azuma

**Affiliations:** 1 Department of Radiology, Osaka Medical College, Takatsuki, Japan; 2 Department of Pathology, Osaka Medical College, Takatsuki, Japan; 3 Department of Urology, Osaka Medical College, Takatsuki, Japan

## Abstract

A 68-year-old male with dementia presented with gross hematuria. On plain CT, a mass was found at the base of the prostate with intravesical protrusion. On MRI, the mass was well-circumscribed and showed slight hyperintensity compared to the skeletal muscle on *T*_1_ weighted imaging and high intensity on *T*_2_ weighted imaging. On dynamic study, the tumor showed mild enhancement in the early phase and increased enhancement in the delayed phase, and the mass appeared continuous with the prostatic urethra. On follow-up MRI at approximately 10 months, the mass had increased in size. Pathologically, the tumor was located in the muscularis of the prostatic urethra and consisted of spindle cells with fascicular and storiform patterns of growth, and exhibited strong diffuse expression of CD34. The tumor was hypercellular, and a significant number of mitoses were observed. Therefore, this tumor was diagnosed as malignant solitary fibrous tumor (SFT) of the prostatic urethra.

## INTRODUCTION

 Solitary fibrous tumor (SFT) is a rare tumor that was first described in the pleura. The incidence is approximately 2.8 per 1,00,000 in pleural SFTs.^1^ Although it is a recognized tumor of the pleura, SFT is thought to be mesenchymal in origin and could arise at virtually any site of the body. In recent years, several reports on extrapleural SFTs have been published, and it has been reported that extrapleural SFTs are more common than pleural SFTs.^2, 3^ This tumor type is generally benign, and reports on malignant cases are limited. Here, we report a case of malignant SFT that arose from the prostatic urethra and present the MRI and histological findings.

## CASE REPORT

**Figure 1.**
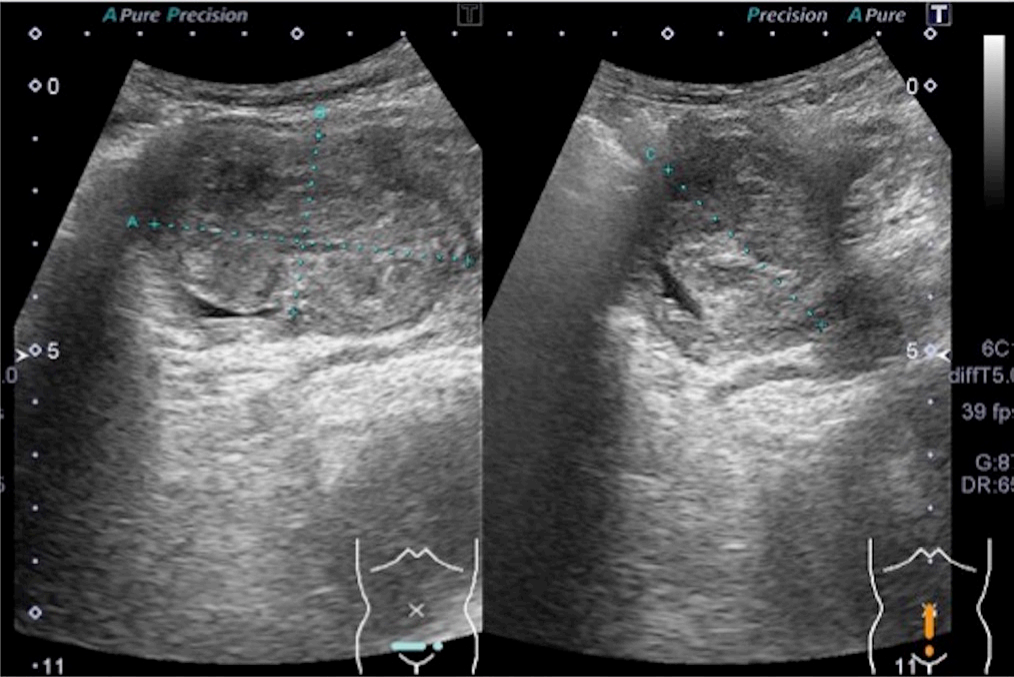
On abdominal ultrasound, a round slightly hyperechoic mass was seen in the bladder.

**Figure 2.**
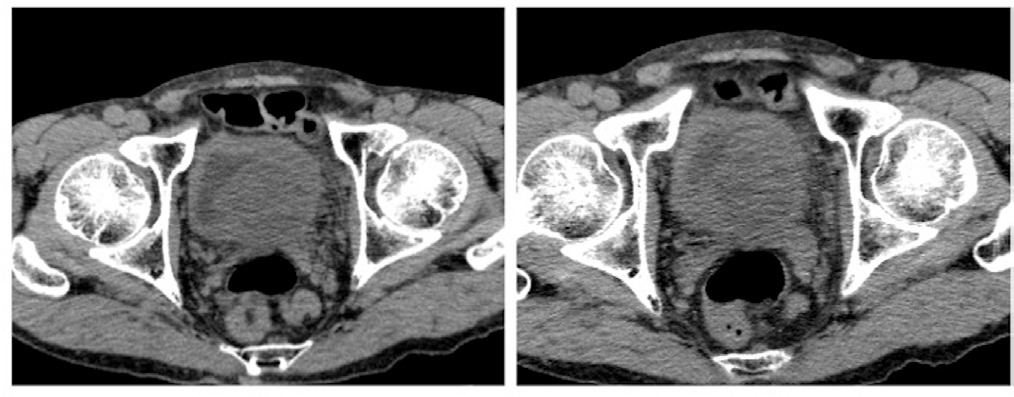
On plain axial CT images, a 4 × 3 cm mass was found at the left side of the base of the prostate with intravesical protrusion. The mass showed isodensity compared to the skeletal muscle.

**Figure 3.**
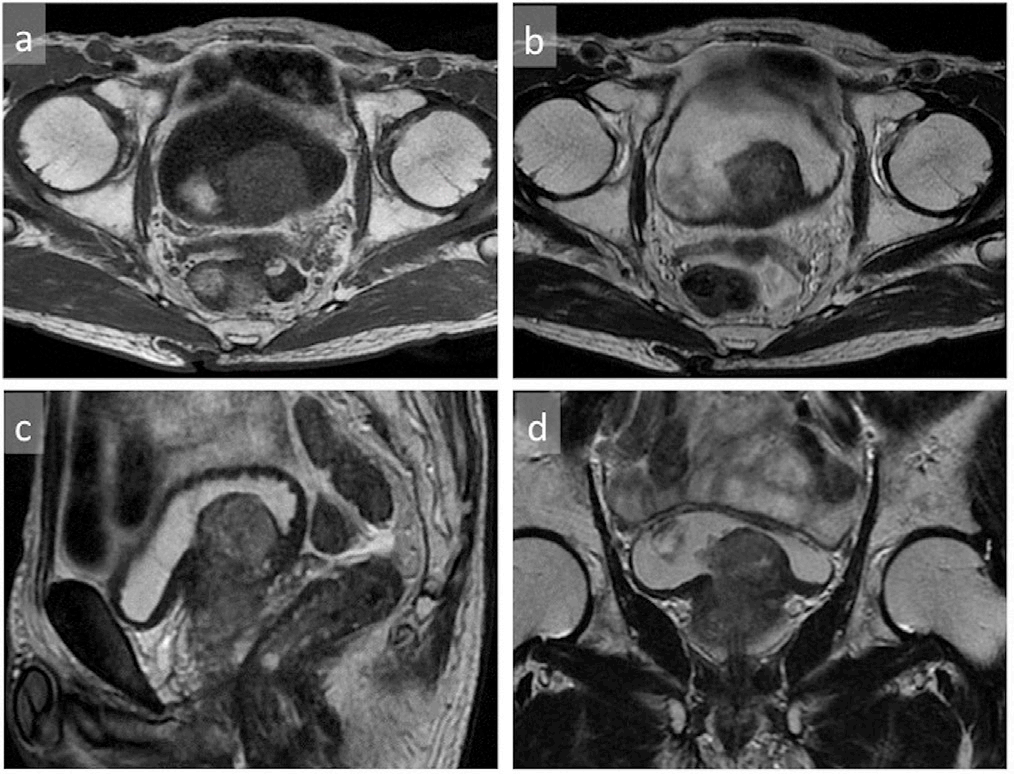
Axial T1WI (a), T2WI (b), coronal T2WI (c) and sagittal T2WI (d) showed a well-circumscribed mass at the base of the prostate. T1WI, *T*_1_ weighted imaging; T2WI, *T*_2_ weighted imaging.

**Figure 4.**
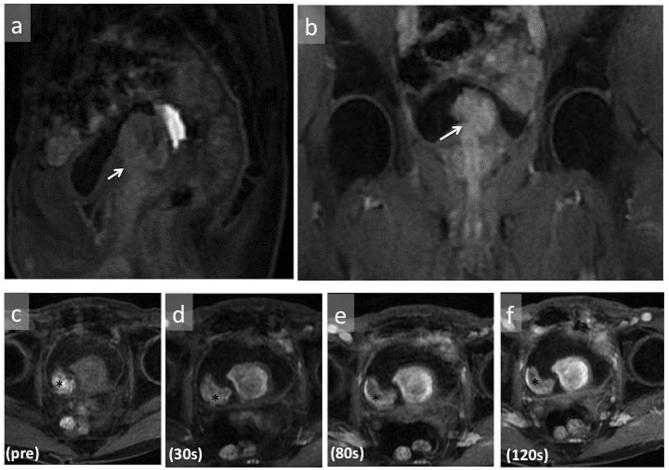
On sagittal (a) and coronal (b) gadolinium-enhanced fat-suppressed T1WI, the tumor was continuous with the prostatic urethra (arrows). The mass showed prolonged enhancement on dynamic gadolinium-enhanced fat-suppressed T1WI (c, d, e, f). A hematoma was seen on the right side of the tumor showing high intensity (*). T1WI, *T*_1_ weighted imaging.

**Figure 5.**
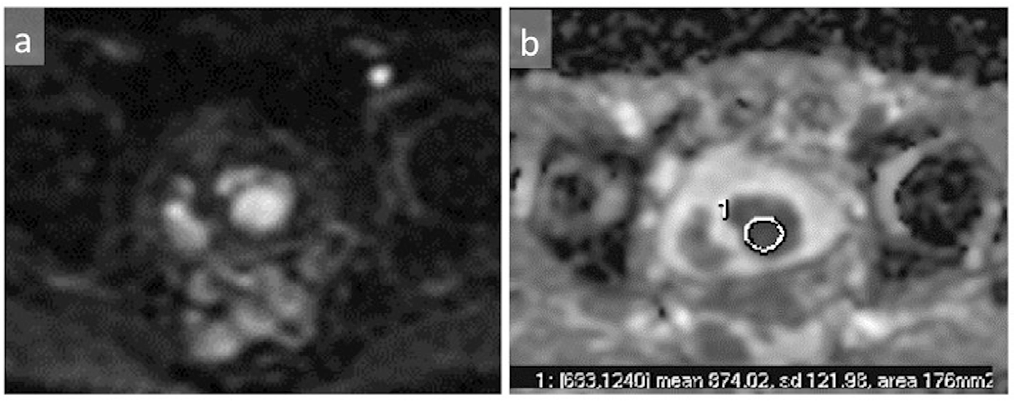
On axial DWI, the mass exhibited high signal intensity (a). A hematoma was seen on the right side of the tumor showing high signal intensity. The ADC value of the mass was decreased (b). ADC, apparent diffusion coefficient; DWI, diffusion-weighted imaging.

 A male in his 60s with dementia suffering from urinary frequency newly presented with gross hematuria. The serum prostatic specific antigen level was within the normal limits. On abdominal ultrasound, there was a round slightly hyperechoic mass in the bladder (Figure 1). On plain CT, a 4 × 3 cm mass and intravesical protrusion were observed at the left side of the base of the prostate (Figure 2). The mass showed isodensity with the skeletal muscle. On MRI, the mass was well-circumscribed and showed slight hyperintensity compared to the skeletal muscle on *T*_1_ weighted imaging (T1WI) and high signal intensity on *T*_2_ weighted imaging (T2WI) (Figure 3). On dynamic gadolinium-enhanced fat-suppressed T1WI, the mass appeared to be continuous with the prostatic urethra, and showed mild enhancement in the early phase and increased enhancement in the delayed phase (Figure 4). On diffusion-weighted imaging (DWI) at a b-factor of 800 s mm^−^^2^, the tumor showed homogeneously high signal intensity and the ADC (apparent diffusion coefficient) value of the tumor was 0.75–1.00 × 10^–3^ mm^2^ s^−1^ (Figure 5).

**Figure 6.**
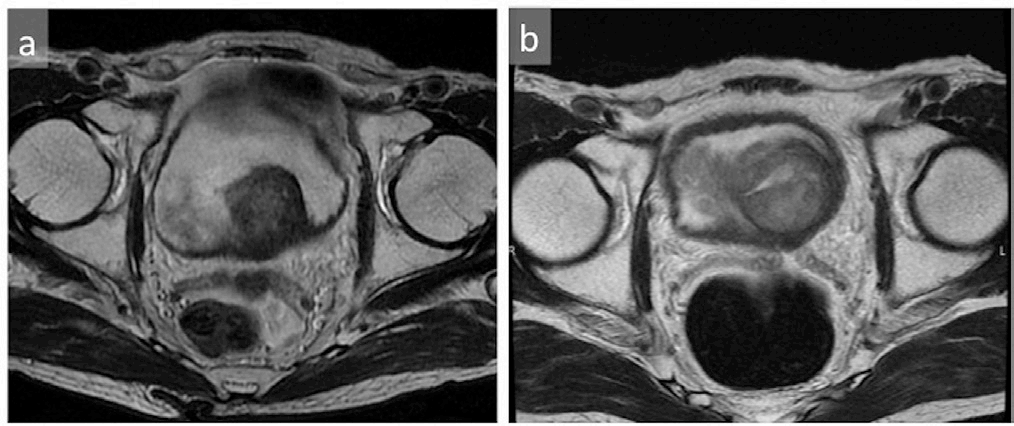
Follow-up MRI enlargement of the mass was seen on follow-up MRI; initial T2WI (a), follow-up T2WI at 10 months (b). T2WI, *T*_2_ weighted imaging.

 Possible radiological differential diagnoses for the mass were stromal tumor of uncertain malignant potential (STUMP) of the prostate, pheochromocytoma of the bladder, leiomyoma/sarcoma, urethral cancer, bladder cancer, prostatic cancer and benign prostatic hyperplasia. Prostatic needle biopsy revealed stromal spindle cells with no mitosis. As there were benign entities among the radiological differential diagnoses and the biopsy revealed no malignancy, conservative management was adopted. Flexible cystoscopy at 9 months revealed a mass at the trigone of the bladder, which appeared continuous with the prostatic urethra. Biopsy revealed fibroblast-like short spindle cells with no evidence of malignancy. On follow-up MRI at approximately 10 months, the mass had increased in size and measured 6 × 5 cm (Figure 6). Prostatic needle biopsy was performed again and revealed tumor cells with round and short spindle-shaped nuclei with some mitoses. These findings favored a malignant lesion, possibly STUMP or stromal sarcoma, and therefore resection of the mass was planned.

**Figure 7.**
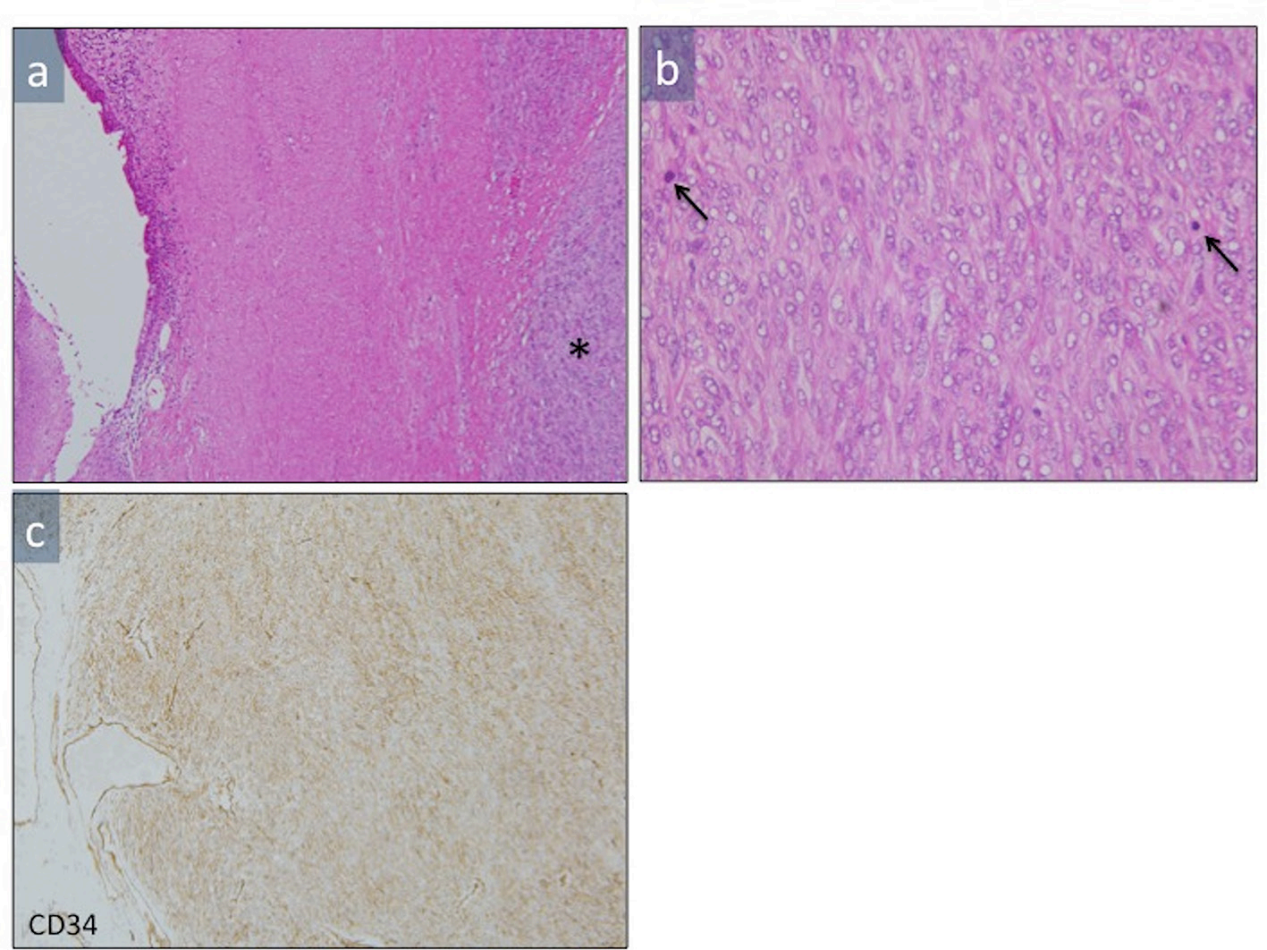
Microscopic appearance of the tumor (a): A low-power view of hematoxylin and eosin-stained sections showed the tumor in the muscularis of the urethra/bladder (*). (b): A high-power view of a hematoxylin and eosin-stained section showing that the tumor consisted of spindle cells with fascicular and storiform patterns of growth. A significant number of mitoses (arrows) were seen in the tumor. (c): The tumor exhibited diffuse and strong expression of CD34.

 Pathologically, the tumor was found in the muscularis of the prostatic urethra or the bladder. The tumor consisted of spindle cells with fascicular and storiform patterns of growth, and mucinous degeneration and some necrosis were observed in the background. The tumor was hypercellular, and a significant number of mitoses (more than 10 in 10 high-power fields) were present. Immunohistochemical analysis revealed diffuse expression of CD34. Therefore, a diagnosis of malignant SFT was made. The above findings are shown in Figure 7. On follow-up CT at 54 months, local recurrence in the pelvis and multiple lung metastases were observed. Currently, the patient is under palliative care.

## DISCUSSION

 SFT was first described by Klemperer and Rabin in the 1930s as a mesothelial tumor that arises from the pleura.^4^ A decade later, Stout and Murray described hemangiopericytoma, the histopathological pattern of which is shared with those of various tumors, including SFTs.^5^ As SFT was initially described in the pleura, they have been considered mesothelial tumors. Due to recent advances in our understanding of the pathogenesis of SFTs, it is currently believed that they are pathologically diverse and ubiquitous neoplasms derived from CD34-positive mesenchymal fibroblastic or myofibroblastic cells.^6, 7^ It has been reported that 16% of SFTs are found in the pelvic region, most frequently in the prostate.^8^ SFTs are generally benign tumors occurring in middle-aged adults, and extrapleural SFTs have a male to female distribution of 1.2:1.^9^ Malignant SFTs are rare and there are no established criteria for diagnosis of malignant SFTs but they are commonly evaluated based on the criteria advocated by England et al.^1^ The characteristics of possibly malignant tumors are large tumors (greater than 10 cm in diameter) with hypercellularity, nuclear atypia, mitoses (>4 in 10 high-power fields) and infiltrative margins. Okada et al reported that atypia was frequently observed in tumors with higher cellularity, which was independent of the tumor size and histological appearance.^10^ In addition, proteins related to malignancy and proliferative activity, such as p53, ki-67 and PCNA, are more frequently observed in tumors with higher cellularity, and of these, p53 is thought to be a prognostic factor.^11, 12^ The most frequently reported sites of metastases of pleural SFTs are the lungs and the pleura; other sites include the brain and the liver. Metastasis has been observed a long time after initial resection, and histological malignant transformations are observed at recurrence.^13–16^

 Imaging finding of SFT indicate a well-circumscribed round or ovoid enhancing mass. On ultrasound, the lesions are reported to be generally hypoechoic but can be heterogeneous with probable presence of myxoid degeneration.^17^ Our case was slightly hyperechoic, and we speculated that variation in the constituents can yield different echogenicity. On plain CT, pleural SFTs often show isodensity with some hypodensity, and they demonstrate mild or marked enhancement on contrast-enhanced CT. It has been reported that the isodense areas on plain CT correspond to hypercellular areas and capillary networks. On contrast-enhanced CT, areas with marked enhancement are correlated with hypervascular areas, areas with intermediate enhancement are correlated with hypercellular areas and patchy or linear hypodense areas are correlated with cystic degeneration, necrosis, or myxoid changes.^18^ On T2WI MRI, some SFTs exhibit mixed hypointensity or hyperintensity. The variation in signal intensity is thought to depend upon tumor components where collagen-rich areas correspond to hypointense areas, while vascular and hypercellular areas correspond to hyperintense areas.^19^ On dynamic enhancement studies, prolonged and delayed enhancement is reported and prolonged enhancement is thought to correspond to abundant hypercellular areas, while delayed enhancement is correlated with collagen-rich areas.^20–22^ On DWI, the ADC value and ADC map were reported to be useful for identifying malignant transformation, with ADC value in the malignant area of 1.12–1.19 × 10^–3^ mm^2^ s^−1^.^23^ In our case, pathologically the mass was hypercellular and its prolonged enhancement was consistent with previous reports. In addition, its ADC value was low as to the reported area with malignant transformation.

 In terms of a round and well-circumscribed mass, prostatic STUMP, leiomyoma/sarcoma and bladder pheochromocytoma are feasible differential diagnoses, and it was impossible to distinguish this case from these entities based on imaging findings alone. Lesions in the base of the prostate are known to mimic bladder-occupying lesions, and it was difficult to identify the exact origin of the tumor.^24^ We believe that our case was SFT originating in the prostatic urethra as the tumor was continuous with the urethra on imaging studies and the same findings were seen on cystoscopy. Moreover, pathologically the tumor was present in the muscularis of the prostatic urethra/bladder. Nonetheless, the mass was relatively large and the possibility that the tumor originated in the bladder or the prostate with secondary extension to the urethra could not be excluded. In the literature, we found one case report of SFT in the urethra of a female patient. This report describes a mobile perineal mass, which was attached to the posterior lip of the urethra.^25^ In our case, the mass protruded to the opposite end, but it appeared to be somewhat similar in morphology.

Of note, conservative management was opted in this case after targeted biopsy yielded no malignancy, but the MRI findings were not consistent with a definitely benign lesion and therefore a second biopsy should have been planned soon afterwards.

 Surgical resection is the treatment of choice for SFT, and the most important prognostic factor is thought to be complete resection with clear margins.^1^ Gold et al reported that extrapleural SFTs have a slightly higher risk of local recurrence than pleural SFTs.^26^ They speculated that this is because of the difficulty in securing wide surgical margins during resection of extrapleural SFTs due to their anatomical location. When the tumors are not surgically resectable or there are metastases, chemotherapy and/or radiotherapy should be considered as palliative treatment. However, the data to establish an evidence-based treatment for advanced SFT are still limited.

 In conclusion, we presented a case of malignant urethral SFT with ultrasound, CT and MRI findings.

## LEARNING POINTS

1. A case of urethral malignant SFT: A T2WI high-intensity round mass appearing to protrude from the urethral opening into the bladder. It showed prolonged enhancement on dynamic gadolinium-enhanced T1WI and high intensity on DWI with an ADC value of 0.75–1.00 × 10^–3^ mm^2^ s^−1^.
2. SFT can arise from virtually any site of the body. If the diagnosis of a well-circumscribed mass with enhancement is in doubt, SFT should be considered as a differential diagnosis.
3. A mass arising in the area around the base of the prostate can protrude into the bladder making local diagnosis difficult.
